# The effect of fluoxetine on astrocyte autophagy flux and injured mitochondria clearance in a mouse model of depression

**DOI:** 10.1038/s41419-019-1813-9

**Published:** 2019-08-02

**Authors:** Xiaodong Shu, Yiming Sun, Xiyang Sun, Yuanzhang Zhou, Yaqi Bian, Zhaoma Shu, Jianhua Ding, Ming Lu, Gang Hu

**Affiliations:** 1 Jiangsu Key Laboratory of Neurodegeneration, Department of Pharmacology, Nanjing Medical University, 101 Longmian Avenue, Nanjing, 211166 Jiangsu China; 2 Department of Pharmacology, Nanjing University of Chinese Medicine, 138 Xianlin Avenue, Nanjing, 210023 Jiangsu China; 3 Neuroprotective Drug Discovery Key Laboratory, Department of Pharmacology, Nanjing Medical University, 101 Longmian Avenue, Nanjing, 211166 Jiangsu China

**Keywords:** Astrocyte, Depression

## Abstract

Although multiple hypotheses had been proposed to clarify the causes of depression, the accurate pathogenesis and effective treatment of depression still need to be solved. Pathological change of astrocytes has been recognized to play a pivotal role in depression. Fluoxetine is the first selective serotonin reuptake inhibitor, however, the underlying mechanisms of fluoxetine are incompletely excavated. Emerging evidence shows that fluoxetine promotes autophagic processes in tumor cells. However, whether astrocytic autophagy gets involved in the cytoprotection of fluoxetine on astrocytes in depression treatment remains unexplored. Here we prepared chronic mild stress (CMS)-induced mouse model and treated mice with fluoxetine (10 mg/kg) for 4 weeks to determine the correlation between proautophagic effect of fluoxetine and astrocyte protection in depression. Primary hippocampal astrocytes were cultured to investigate the potential mechanism of fluoxetine in regulating astrocyte autophagy. We found that fluoxetine (10 mg/kg) treatment promoted autophagosome formation and increased clearance of injured mitochondria, consequently protected astrocytes in CMS model mice. Fluoxetine (10 μM) could also promote the autophagic flux unblocked via enhancing fusion of autophagosomes with lysosomes in primary astrocytes. Moreover, fluoxetine promoted mitophagy by increased colocalization of autophagosomes and mitochondria, eliminating damaged mitochondria in corticosterone-treated astrocytes. Further in vitro study showed that p53 presence is required for fluoxetine activated autophagy flux and fluoxetine promotes astrocytic autophagy in a p53-dependent mechanism. Collectively, this work gives us insights into a novel approach to treat depression depending on astrocytes, and provides a promising molecular target for the development of antidepressant drugs besides regulating neurotransmitters.

## Introduction

Depression is a devastating psychiatric disease characterized by anhedonia, low motivation, pessimism, and self-accusation^1^. At present, more than 120,000,000 people live under the shadow of depression and patients suffering from major depressive disorder would have the propensity to suicide^2^. There are ~850,000 cases of tragedies taking place every year^3^, while there are few rapid, robust, and sustained antidepressants used clinically. Emerging evidences reveal that the abnormal excitatory synapse^4^, overactivated microglia^5^, and neuroimmune and neuroinflammatory reactions^6^ are also involved in the onset of depression. Recent report holds the perspective that neuronal autophagy plays a potential role in the pathophysiology and treatment of depression^7^ and autophagy has been recognized as one of the possible mechanisms for newly developed antidepressants^8^.

Autophagy, including mitophagy, is a tightly modulated cellular degradation pathway by which defective proteins, impaired organelles, and other cellular constituents are sequestered in autophagosomes and delivered to lysosomes for degradation^9^. Moderate autophagy is generally considered as a self-protection mechanism against cellular damage caused by intracellular and extracellular stress^10^. Astrocyte, the cell type with the largest number in central nervous system, whose pathological damage is involved in many neurological diseases^11^. It has been known that decrease in cellular number and volume of astrocytes in cortex and hippocampus plays an important role in the pathogenesis of major depression^12^. Generally, the energy production of astrocytes is largely based on mitochondrial oxidative metabolism^13^ in response to neuronal activity. Therefore, mitophagy as a selective autophagy may play a crucial role in determining the fate of astrocytes in depression. So far, however, it remains unclear the significance of astrocytic autophagy in the pathogenesis and treatment of depression.

Fluoxetine is the first selective serotonin reuptake inhibitor (SSRI), which is approved for the clinical treatment of depression^14^. Fluoxetine improves patients’ emotion via upregulating the concentration of synaptic cleft 5-HT^15–17^. Previous studies indicated that fluoxetine exerts neuroprotective^18^, anti-inflammatory^19^, and antitumor^20^ effects. Beyond its influence on the monoaminergic neurotransmission, fluoxetine also exhibits the potential impacts on autophagic processes and functions as a novel proautophagic agent^21,22^. Gassen et al. reported that fluoxetine treatment evoked a response of the autophagic markers beclin-1, Atg12, pAkt, and LC3B-II/I in primary astrocyte cell culture^23^. Furthermore, Gulbins et al. found that widely used antidepressants such as amitriptyline and fluoxetine induce autophagy in hippocampal neurons via the slow accumulation of sphingomyelin in lysosomes and Golgi membranes and of ceramide in the endoplasmic reticulum^24^. Therefore, autophagy modulation has been linked to the action of different antidepressants^25^. However, the involvements of fluoxetine-regulated autophagy in astrocyte pathology in vivo and in depression-like behaviors are not yet clarified. In the present study, we prepared chronic mild stress (CMS)-induced mouse model of depression so as to determine the correlation between proautophagic effect of fluoxetine and astrocyte protection in depression.

## Materials and methods

All experiments were carried out according to the National Institutes of Health Guidelines for the Care and Use of Laboratory Animals, and all animals were treated in strict accordance with protocols approved by the Institutional Animal Care and Use Committee of Nanjing Medical University. All applicable international, national, and/or institutional guidelines for the care and use of animals were followed.

### Animals

Male C57/BL6J mice weighing 18–25 g were purchased from the Comparative Medicine Centre of Yangzhou University. Each mouse was housed in a single cage with free access to food and water and was divided into different groups randomly. Room temperature was maintained at 22 ± 2 °C.

### Fluoxetine treatments and CMS paradigm

Fluoxetines were prepared in saline, and given at a dose of 10 mg/kg for 4 weeks. Each mouse was caged singly and adapted to the environment for 3 days, then they were pretrained to drink sucrose solution. CMS was consistent with the details described previously. The CMS paradigm consists of various mild stressors, such as food and water deprivation, inversion of day/night light cycle, 45° tilted cage, restraint, pairing with another stressed mouse, soiled cage bedding, etc. Each of these stressors was guaranteed not to be applied in consecutive days. Control mice were placed in a suitable environment separating from the stressed animals.

### Sucrose preference test

Mice were tested to drink from two bottles for 12 h (from 09:00 to 21:00), one bottle contained sucrose solution (1%) and the other contained tap water. In order to prevent possible side preference in the test, the positions of the bottles were exchanged after 6 h. The animals were deprived of water before the test. The consumption of tap water, sucrose solution, and total intake of liquids were assessed by weighing the bottles in the control and experimental groups. The ratio of the consumed sucrose solution vs. the total amount of liquid intake was calculated as the preference for sucrose.

### Forced swimming test (FST)

The FST employed was essentially similar to that described elsewhere. Briefly, mice were individually placed in a transparent 2 L glass cylinder (19 cm tall) filled with water at 23 °C, to a depth of 13 cm, and left there for 6 min. A mouse was judged to be immobile when it floated in an upright position, and made only small movements to keep its head above water. The duration of immobility was recorded during the last 4 min of the 6-min testing period by TailSuspScan^TM^ (Clever Sys Inc., VA, USA).

### Tail suspension test (TST)

The TST is one of the most widely used models for assessing antidepressant-like activity in mice. In this experiment, mice were individually suspended by the distal portion of their tails with adhesive tape for a period of 6 min (30 cm from the floor) in a visually isolated area. The time of immobility of the tail-suspended mice during the last 4 min was measured with TailSuspScan^TM^ (Clever Sys Inc., VA, USA).

### Corticosterone content determination

Brain tissues from mice in each group were homogenized with lysis buffer to extract total protein. The homogenate was centrifuged at 21,000 *g* at 4 °C for 30 min. Peripheral blood from mice in each group at room temperature for 2 h, centrifuged at 3000 *g* at 4 °C for 10 min, then collected the supernatant. The concentration of CORT in vivo was measured by mouse CORT ELISA kits (Jin Yibai Biological Technology, JEB-12812) according to the manufacturer’s instructions.

### Transmission electron microscopic (TEM) analysis

Mice were perfused with 2.5% glutaraldehyde and 2% paraformaldehyde. A small portion (~1 mm^3^) of the hippocampus was sectioned and incubated for 2 h at 4 °C in the same fixative. Specimens were postfixed in 1% osmium tetroxide, stained in aqueous uranyl acetate, and then dehydrated and embedded in epoxy resin. Ultrathin sections were stained using lead citrate and examined with transmission electron microscope (JEM-1010, Tokyo, Japan). All experiments and photographs of TEM were supported by the grant from the Center of Forecasting and Analysis of Nanjing Medical University.

### Western blot analysis

Proteins extracts were electrophoresed and incubated with corresponding specific antibodies against LC3B (1:1000, Cell Signaling Technology, #2775), p62 (1:1000, Cell Signaling Technology, #5114), phospho-mTOR (Ser2448, 1:1000, SAB, #11221-2), mTOR (1:1000, SAB, #21214-2), Atg7 (1:1000, ABGENT, AP1813a), beclin-1 (1:1000, Cell Signaling Technology, #3738), p53 (1:1000, Cell Signaling Technology, 2524), and β-actin (1:4000, Sigma) overnight and enveloped with appropriate secondary antibodies for 1 h, and then developed.

### mTagRFP-Wasabi-LC3 plasmid transfection and fluorescence puncta counting

Primary astrocytes were seeded onto the glass coverslips in the 24-well plates. After the density reached 70–80%, cells were transfected with 0.5 μg mTag-Wasabi-LC3 plasmid and manipulated in accordance with the instruction of Lipofectamine^TM^ 3000 (Invitrogen, USA). Six hours after transfection, reagents were replaced by nutrient medium containing 10% FBS and cultured overnight. LC3 fluorescent spots were captured by a fluorescence microscope. Counting the vesicles of autophagosomes and autolysosomes was performed manually by cell counter plugin in Image J. Five random fields were selected for each sample and specific puncta with visible fluorescence intensity were counted. Results were based on three independent experiments.

### Immunofluorescent staining

For labeling GFAP and LC3, cells were incubated with mouse anti-GFAP (1:500, Millipore, #MAB360) and rabbit anti-LC3 (1:400, Cell Signaling Technology, #2775) overnight. Then, incubated with Alexa Fluor^®^ secondary antibodies for 1 h, cells were observed under a fluorescence microscope. For labeling autophagosomes and mitochondria, primary astrocytes were plated onto poly-l-lysine-handled glass coverslips and transfected with GFP-LC3. After treated with fluoxetine and corticosterone (10 μM) for 24 h, cells were loaded with 100 nM MitoTracker Deep Red (Molecular Probes, M22426, Invitrogen) at 37 °C for 30 min and then washed with prewarmed D-hank’s for three times followed by fixation with 4% paraformaldehyde. Glass coverslips were observed under a stereo microscope.

### Flow cytometry assay

Cultured cells were collected after stimuli and resuspended in PBS. Cells were fixed with 4% formaldehyde at room temperature for 15 min, and washed with enough PBS. Slowly add ice-cold 90% methanol to the precooled cells by gentle vortex mixing to permeabilize the cells, followed by incubation on ice for 30 min. Centrifugation and wash with enough PBS to remove methanol. Next, the cells were incubated with the LC3B antibody (1:50) for 1 h at room temperature. The cells were then washed and incubated with Alexa Fluor 488-conjugated goat anti-rabbit (Invitrogen, A11008; 1:1000), incubated for 30 min at room temperature. Resuspend the cells in PBS and analyze with a flow cytometer. For MitoSOX detection, MitoSOX^TM^ (Molecular Probes, M36008, Invitrogen) was used to stain cells for 10–30 min at 37 °C, protected from light. Afterward, cells were washed gently three times with prewarmed D-hank’s and resuspended, then detected on flow cytometer (Guava, Easycyte^TM^ 8, Millipore).

### Mitochondria and lysosome staining

MitoTracker Green (Molecular Probes, M7514, Invitrogen) and LysoTracker Red (Molecular Probes, L7528, Invitrogen) were used to label the mitochondria and lysosome in astrocytes. After the corresponding management, these two dyes were added in the DMEM (Gibco) in a proportion of 1/500 simultaneously. After 30 min, we discarded the medium and washed cells twice with prewarmed D-hank’s. Afterward, cells were fixed with 4% paraformaldehyde and then observed under the stereo microscope (Olympus).

### Mitochondrial protein extraction

We discarded the supernatants and added 1 mL mitochondrial isolating reagents (Beyotime), which were added PMSF (1 mM) several minutes ago. Cells were ice-bathed for 10–15 min followed by a 10–30-time homogenization, then they were centrifuged at 600 *g* for 10 min at 4 °C. We transferred the supernatants into another EP pipe carefully, which then were centrifuged at 11,000 *g* for 10 min at 4 °C and the sediments we got were just the mitochondria. We transferred the supernatants at this time and centrifuged at 12,000 *g* for 10 min at 4 °C and the supernatants we got were the cytoplasmic protein without mitochondria. Afterward, we added 150 μL mitochondrial lysis buffer (containing PMSF) into the isolated mitochondria to split on ice for 30 min. When time was over, samples were centrifuged at 16,000 *g* for 15 min at 4 °C, the supernatants were the mitochondrial proteins.

### Reactive oxygen species (ROS) detection

Cells were pretreated with 3-MA (3-methyladenine, Sigma, m-9281, USA, 5 mM)/CQ (chloroquine diphosphate, Sigma, C6628, USA, 10 μM)/BafA1 (bafilomycin A1, Selleck, S1413, China, 100 nM) for 1 h, followed by fluoxetine (10 μM) and corticosterone (10 μM) incubation for 24 h. Supernatants were discarded and H_2_DCF-DA (20 μM) was added to stain for 30 min at 37 °C. Cells were washed twice with D-hank’s solution, then fixated with 4% paraformaldehyde for 15 min and observed under a fluorescence microscope (Nikon).

### Cell viability assay

Cells were cultured in the 96-well plate and were pretreated with 3-MA (5 mM)/CQ (10 μM)/BafA1 (100 nM) for 1 h, followed by fluoxetine (10 μM) for another 1 h. The high concentrations (300, 600, and 1200 μM) of CORT were used only to induce the death of astrocytes. After treatment, we discarded the medium and performed according to the instruction of EnoGeneCell^TM^ Counting Kit-8 (CCK-8). Ten microliters CCK-8 solution was added in one well (no bubble) and the plate was put in the incubator for about 3 h. We measured the wavelength of each well at 450 nm by microplate reader when time was over.

### Statistical analysis

All values are expressed as the mean ± SD. Differences among means were analyzed using one-way analysis of variance with treatments as independent factors. *p* < 0.05 was defined as significant.

## Results

### Fluoxetine attenuated the decrease in number of astrocytes in CMS model mice

**Fig. 1:**
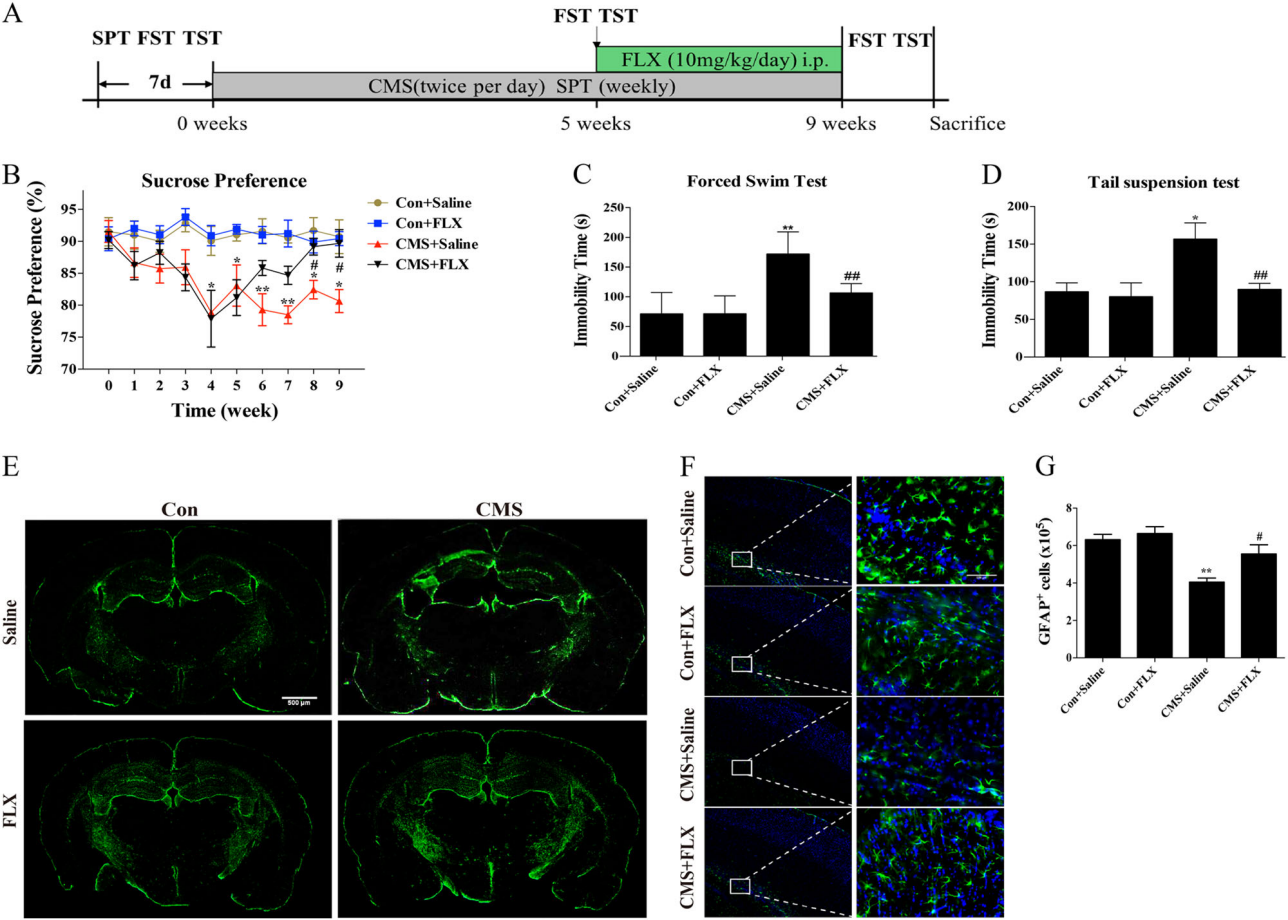
Fluoxetine improved behavioral symptoms and alleviated astrocyte damage in CMS model mice. **a** The program of CMS model preparation and drug administration in the present study. **b** Percentage of sucrose preference in control and CMS groups in the presence or absence of fluoxetine treatment. *n* = 15 in each group. **c** The immobility time in forced swimming test and in **d** tail suspension test. *n* = 15. **e** Immunofluorescence of GFAP^+^ astrocytes in whole mouse brain slice, scale bar: 500 μm. **f** Immunofluorescence of GFAP^+^ cells in mouse hippocampus, scale bar: 100 μm and **g** stereology quantitative counting of GFAP^+^ cells in dentate gyrus of hippocampus. *n* = 6, data are expressed as mean ± SD. *p < 0.05, **p < 0.01 vs. Con group, ^#^*p* < 0.05 vs. CMS group

As shown in Fig. 1a, mice were subjected to CMS for about 5 weeks to establish depression model and followed by treatment with fluoxetine (10 mg/kg/day, i.p) for 4 weeks in the present study. After 5 weeks of CMS modeling, the percent of sucrose preference significantly decreased compared with those in control groups (Fig. 1b). Fluoxetine administration increased the sucrose consumption gradually and showed significant difference at the end of 8- and 9-week treatment compared with those in the saline-treated CMS group (Fig. 1b). Consistently, fluoxetine administration for 4 weeks significantly reduced the immobility time of CMS mice in FST and TST (Fig. 1c, d) and extended the swimming time and climbing time in FST (Supplementary Fig. 1). Furthermore, fluoxetine treatment also suppressed CMS modeling-induced elevation of corticosterone content in both of mouse hippocampus and plasma (Supplementary Fig. 2). Notably, it was found that the number of GFAP^+^ astrocytes decreased in hippocampus and cortex of CMS mice, while fluoxetine treatment attenuated the loss of astrocytes in CMS model (Fig. 1e). Immunohistochemistry and quantitative counting result showed fluoxetine rescued GFAP^+^ cell number in hippocampus of CMS mice compared with that in saline-treated CMS group (Fig. 1f, g). These findings suggest that ameliorating astrocyte pathological damage may contribute to the antidepressant effect of fluoxetine in CMS mouse model.

### Fluoxetine promoted autophagosome formation and ameliorated mitochondrial impairment in the hippocampal astrocytes of CMS mice

**Fig. 2:**
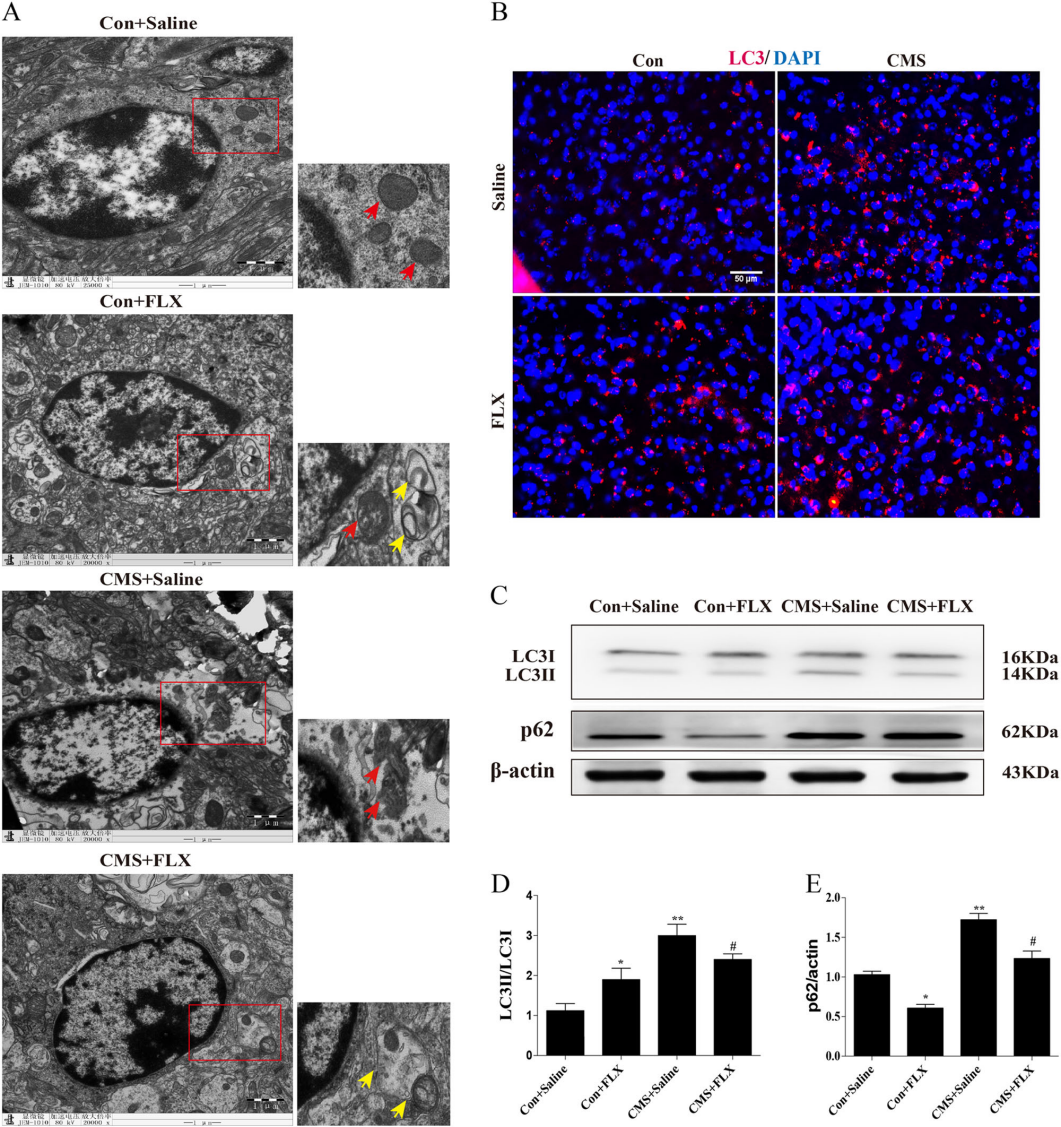
Fluoxetine ameliorated mitochondrial impairment and promoted autophagosome formation in hippocampus of CMS mice. **a** Transmission electron microscope showed that mitochondria were normal in control and fluoxetine-treated group (red arrows). Mitochondria were severely disrupted in CMS group and the disruption was significantly ameliorated in fluoxetine-treated CMS group (red arrows). Several autophagosomes were observed in fluoxetine alone and fluoxetine-treated CMS group (yellow arrows). **b** Immunofluorescence of LC3 protein in mouse brain, scale bar: 50 μm. **c** Representative western blotting of LC3 and p62 expressions, and **d**, **e** quantitative analysis of protein levels. *n* = 5, data are expressed as mean ± SD. *p < 0.05, **p < 0.01, ***p < 0.001 vs. Con, ^#^*p* < 0.05 vs. CMS group

To confirm the protective effect of fluoxetine on hippocampal astrocytes in CMS model and to clarify the potential mechanism, we used TEM analysis to observe the ultrastructure of hippocampal astrocytes in mouse brain slices. Under TEM, astrocytes could be distinguished from other cell types in central nervous system by their oval nucleus, deep chromatin, and filament in cytoplasm. We found mitochondria (red arrows) were disrupted in CMS group while the impairment was alleviated after fluoxetine treatment (Fig. 2a). Most importantly, we also observed several autophagosomes (yellow arrows) in cytoplasm, which located close to mitochondria or even enveloped them in both fluoxetine alone and fluoxetine-treated CMS groups (Fig. 2a). The result of TEM suggests that fluoxetine significantly ameliorates mitochondrial impairment and promotes autophagosome formation in hippocampus of CMS mice. Brain slice immunofluorescence staining also showed that either CMS modeling or fluoxetine treatment increased the expression of LC3 (microtubule-associated protein light-chain 3, Fig. 2b), which is recognized as a ‘receptor'' at the phagophore and interacts with the ‘adaptor'' molecules on the target, such as protein aggregates, mitochondria. To a certain extent, the ratio of LC3-II to LC3-I represents autophagy level. Thus, we further applied western blotting analysis and result showed the rise in ratio of LC3-II to LC3-I in CMS and fluoxetine treatment groups (Fig. 2c, d). Given that LC3 can only reflect the initialization of autophagy flux, we also detected the protein level of p62, which is the best-characterized molecule and degradation product of autophagy^10^. As shown in Fig. 2c, e, hippocampal p62 levels were increased in CMS mice but significantly decreased in fluoxetine-treated mice. Combined with LC3 expression, it implies that autophagic flux is blocked in CMS mice, whereas fluoxetine promotes unobstructed autophagic flux in basic state and CMS model.

### Fluoxetine exerted proautophagic effect in primary cultured mouse astrocytes

**Fig. 3:**
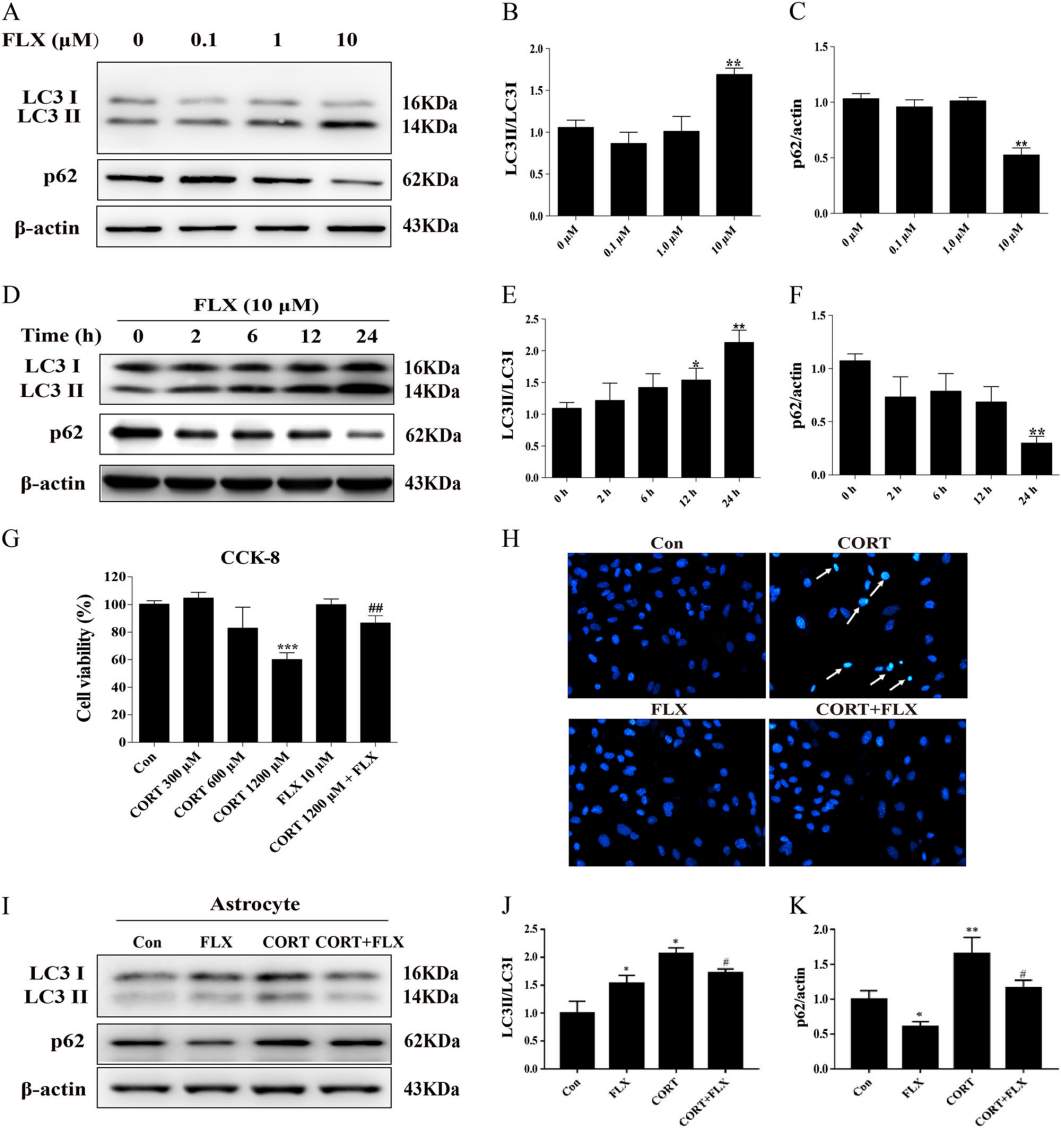
Fluoxetine attenuated corticosterone-induced apoptosis and activated autophagy in primary cultured astrocytes. **a**–**c** Primary astrocytes were subjected to fluoxetine in the concentration of 0.1, 1.0, and 10 μM and **d**–**f** treated with 10 μM fluoxetine for 2, 6, 12, and 24 h. Classic autophagic markers, LC3II/I and p62, were analyzed using western blotting. **g** CCK-8 assay detected the cytoprotection of fluoxetine on corticosterone-inhibited astrocyte survival. **h** Hoechst staining showed the antiapoptotic effect of fluoxetine on corticosterone-induced cell death, at 20× magnification. **i** Representative image and **j**, **k** quantitative analysis of LC3II/I and p62 expression in the treatment of FLX (10 μM) and CORT (1.2 mM). Data are representative of four independent experiments and are expressed as mean ± SD. *p < 0.05, **p < 0.01, and ***p < 0.001 vs. respective control groups

To determine the proautophagy effect of fluoxetine in vitro, mouse hippocampal primary astrocytes were treated with fluoxetine at various concentrations (0.1–10 μM). We next detect classic autophagic markers LC3 and p62 by Western blotting analysis. As shown in Fig. 3a–c, fluoxetine upregulated LC3 expression and decreased p62 level gradually (0.1–10 μM), while had a significant effect at 10 μM. Furthermore, we treated astrocytes with fluoxetine (10 μM) to evaluate the time course of proautophagic effect. We collected astrocytic samples at the time point of 2, 6, 12, and 24 h after treatment, respectively. It was found that fluoxetine (10 μM) exerted the maximal proautophagic effect at the end of 24 h treatment evidenced by increased LC3-II and reduced p62 expressions (Fig. 3d–f). To confirm the protective role of fluoxetine on astrocytes in vitro, we evaluated cell viability by CCK-8 assay. As shown in Fig. 3g, corticosterone at 1.2 mM significantly inhibited cell viability while relatively low concentration had no impact on cell viability. Fluoxetine (10 μM) pretreatment rescued the survival of astrocytes that were injured by corticosterone. Hoechst staining also exhibited the antiapoptotic effect of fluoxetine on corticosterone-induced nuclear pyknosis and cell death (Fig. 3h). These results confirm the protective role of fluoxetine on astrocytes, which is found in CMS mouse model in vivo. Meanwhile, western blotting result showed the rise in ratio of LC3-II to LC3-I in both CORT group and FLX group, indicating a formation of initial autophagosome. However, autophagy substrate p62 levels were increased in CORT group but significantly decreased in FLX group, and fluoxetine pretreatment inhibited corticosterone-induced p62 accumulation (Fig. 3i–k). These results suggest that corticosterone blocks autophagy flux, whereas fluoxetine promotes autophagy flux.

### Fluoxetine promoted autophagic flux of primary astrocytes effectively

**Fig. 4:**
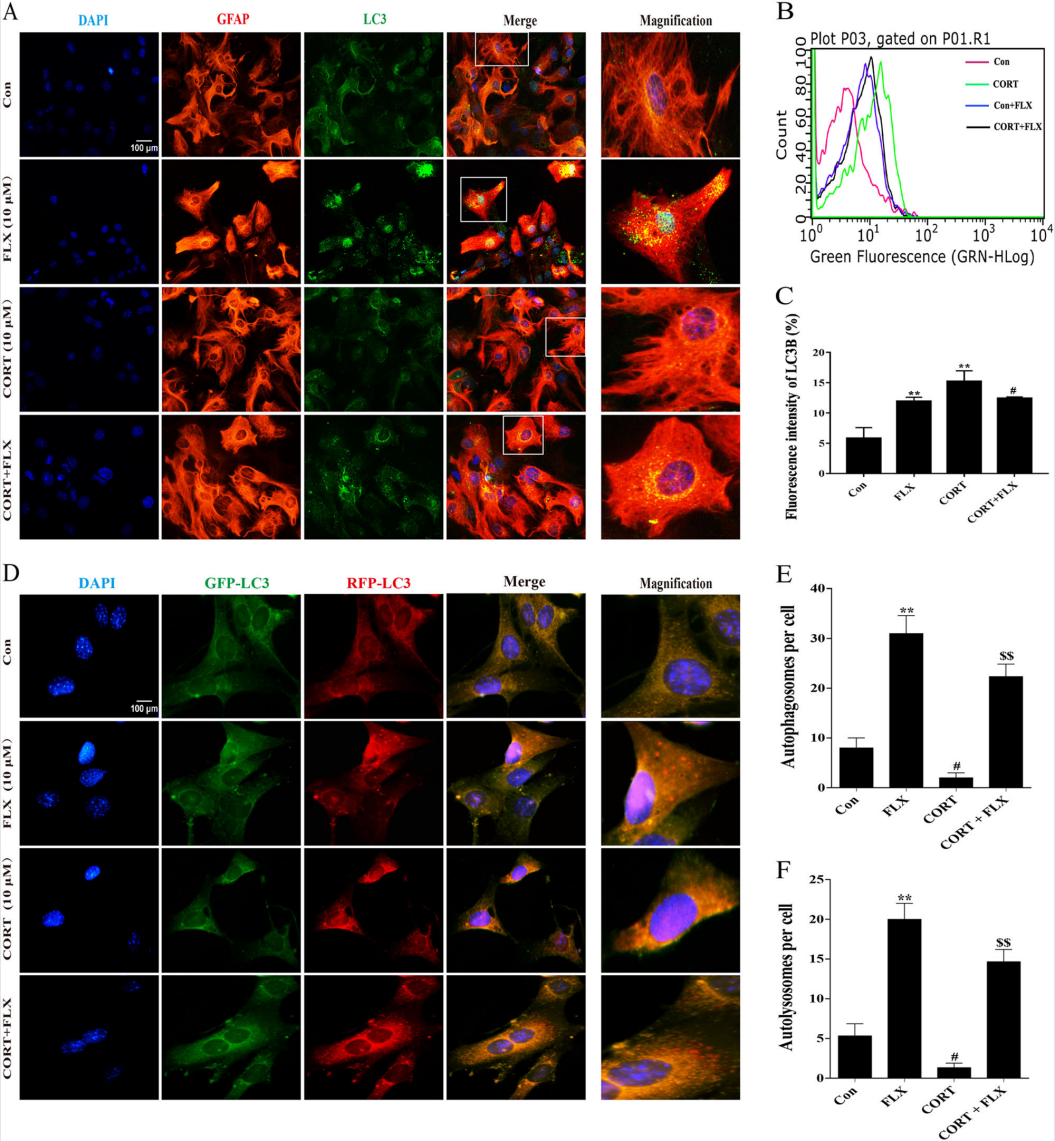
Fluoxetine promotes autophagic flux in primary astrocytes effectively. **a** Primary astrocytes were immunostained with GFAP (green), LC3 (red), and DAPI (blue) simultaneously. LC3 puncta significantly increase after 10 μM fluoxetine treatment. **b**, **c** Quantitative analysis of LC3 puncta immunofluorescence intensity by flow cytometry in each group. **d** Immunofluorescence staining of astrocytes transfected with mTag-Wasabi-LC3 plasmid. A part of GFP-LC3 puncta is degraded in lysosomal acidic environment, while RFP-LC3 is resistant to it. After fluoxetine treatment, puncta of RFP-LC3 are much more than those of GFP-LC3. **e**, **f** Quantitative analysis of autophagosomes and autolysosomes per cell in each group. Scale bar: 100 μm. Data are representative of three independent experiments and are expressed as mean ± SD. *p < 0.05, **p < 0.01 vs. Con group, ^#^*p* < 0.05, ^##^*p* < 0.01 vs. Con group, ^$$^*p* < 0.01 vs. CORT group

Under the condition of autophagosomal accumulation, immunocytochemistry signals will increase and appear puncta due to increased LC3-II, which is localized to autophagic structures^26^. Our results indicated that fluoxetine alone markedly increased the overlay of GFAP (red) and LC3 (green) in astrocyte cytoplasm, while corticosterone decreased LC3 puncta. Pretreatment of fluoxetine also elevated the number of LC3 fluorescent spots in response to corticosterone challenge (Fig. 4a). We next alternatively quantify LC3-positive puncta using flow cytometry. It was found that both FLX and CORT could enhance LC3 immunofluorescence signals (Fig. 4b, c), which is consistent with the result obtained from western blotting in Fig. 3j. The accumulation of autophagosomes may be due to either increased initiation or decreased autophagic completion. In the latter circumstance, the inner membrane-localized LC3-II cannot be degraded and the number of LC3-positive puncta increases as a result of the accumulation of autophagosomes. To distinguish whether the accumulation of autophagosomes is due to decreased fusion of autophagosomes with lysosomes or increased autophagosomal formation, the tfLC3 (tandem fluorescently tagged LC3) method has been developed to solve this problem^27^. In the present study, we thus used mTagRFP-Wasabi-LC3 plasmid to detect autophagic flux. Once the maturation of an autolysosome occurs, only the RFP signal can be exhibited because it is resistant to the lysosomal acidic/proteolytic environment. As shown in Fig. 4d, either fluoxetine or corticosterone enhanced GFP-LC3 expression, suggesting they can both initiate autophagosomal formation. However, corticosterone decreased RFP-LC3 accumulation, while fluoxetine significantly increased RFP-LC3 expression and spot aggregation in the presence and absence of corticosterone stimuli. Quantitative analysis of autophagosomes and autolysosomes supported the immunofluorescence results (Fig. 4e, f). These immunofluorescence images indicate that fluoxetine, contrary to corticosterone, promotes the autophagic flux by increasing fusion of autophagosomes with lysosomes in astrocytes.

### Mitophagy induced by fluoxetine in primary astrocytes

**Fig. 5:**
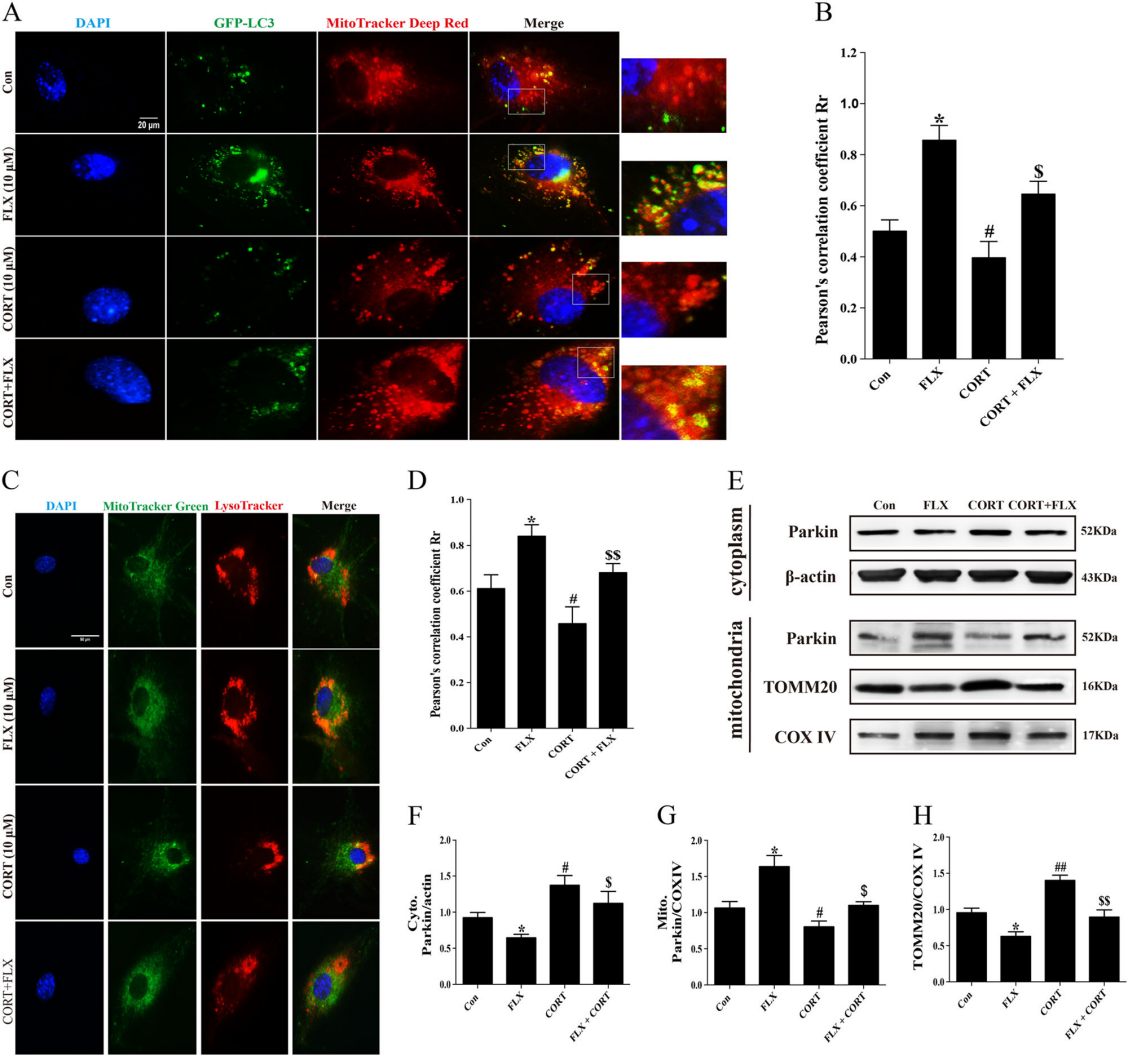
Mitophagy was induced by fluoxetine in primary astrocytes. **a** Immunofluorescence for colocalization of LC3 and mitochondria in primary astrocytes. Cells were treated with 10 μM fluoxetine whose mitochondria were labeled by MitoTracker Deep Red. Each green spot represents one autophagosome. Scale bar: 20 μm. **b** Pearson’s correlation coefficient for colocalization of LC3 and mitochondria. **c** Immunofluorescence for colocalization of mitochondria (MitoTracker Green) and lysosomes (LysoTracker Red) in primary astrocytes. Scale bar: 60 μm. **d** Pearson’s correlation coefficient for colocalization of lysosomes and mitochondria. **e**–**h** Western blotting and quantitative analysis of Parkin and TOMM20 expressed in cytoplasm and mitochondria. Data are representative of four independent experiments and are expressed as mean ± SD. *p < 0.05 vs. Con group, ^#^*p* < 0.05, ^##^*p* < 0.01 vs. Con group^, $^*p* < 0.05, ^$$^*p* < 0.01 vs. CORT group

In the present study, transmission electron microscope results showed the mitochondrial rupture and impairment in hippocampal astrocytes of CMS model mice. Therefore, we colabeled autophagosomes and mitochondria under different conditions to determine the effect of fluoxetine on mitophagy. The representative images displayed that colocalization of autophagosomes and mitochondria was increased after fluoxetine treatment under normal condition and corticosterone stimulation (Fig. 5a, b). Since autophagosomes eventually need to fuse with lysosomes, the colocalization of mitochondria with lysosomes can also alternatively be used to monitor mitophagy. Next, we used MitoTracker Green and LysoTracker Red to visualize the effect of fluoxetine on mitophagy flux. The overlay of mitochondria and lysosomes was significantly increased in fluoxetine-treated alone group and fluoxetine could facilitate mitophagy following incubation of corticosterone (Fig. 5c, d). As we know, Parkin plays a critical role in mitophagy in mammalian cells^28^^,^^29^. TOMM20 is mitochondrial outer membrane protein and can be degraded by E3 ubiquitin ligase Parkin recruited by Pink1. Given these, we further isolated mitochondria of astrocytes and extracted mitochondrial proteins and cytoplasmic proteins without mitochondria. Immunoblot analysis showed that Parkin was translocated from cytoplasm to mitochondria to degrade TOMM20 and induced a decrease in mitochondrial TOMM20 protein level following treatment with fluoxetine (Fig. 5e–h). In contrast, corticosterone inhibited the mitochondrial translocation of Parkin and reduced TOMM20 degradation, disrupting the process of mitophagy. But fluoxetine pretreatment could restore the blockade of mitophagy flux induced by corticosterone (Fig. 5e–h). These results demonstrate that fluoxetine promotes astrocytic mitophagy and the clearance of damaged mitochondria in corticosterone-treated cell model.

### Fluoxetine-induced autophagy in a p53-dependent manner

**Fig. 6:**
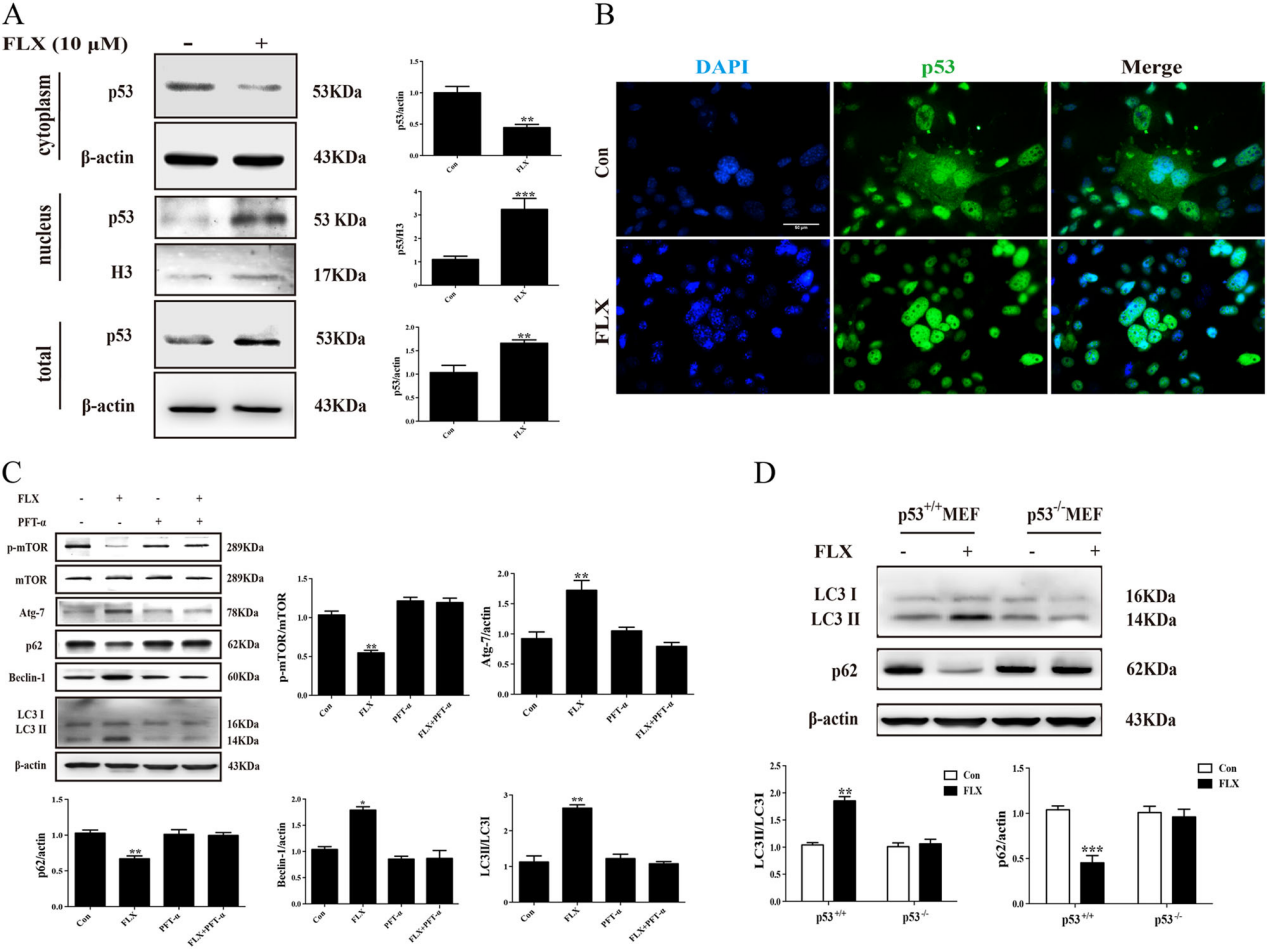
p53 mediated proautophagic effect of Fluoxetine in astrocytes. **a** Representative western blotting and quantitative analysis of p53 trafficking from cytoplasm into nucleus. **b** Immunofluorescence for nuclear translocation of p53 induced by fluoxetine in astrocytes. Scale bar: 50 μm. **c** Western blotting analysis for the effect of p53 inhibitor, PFT-α on the expressions of p-mTOR, Atg7, LC3II/I, p62, and beclin-1 in astrocytes. **d** Western blotting analysis for the effect of fluoxetine on LC3II/I, p62 protein levels in wild-type and p53^−/−^ MEF cells. Data are representative of four independent experiments and are expressed as mean ± SD. *p < 0.05, **p < 0.01 and ***p < 0.001 vs. respective Con groups

Our previous study revealed the effect of fluoxetine on “gatekeeper” p53 expression in neurons^30^. To investigate the detailed mechanism for fluoxetine-induced autophagy in astrocytes, we thus determined the role of p53 in fluoxetine-evoked autophagy flux. As shown in Fig. 6a, fluoxetine (10 μM) markedly enhanced nuclear translocation of p53 evidenced by the decreased p53 level in cytoplasm and the increased p53 level in nucleus. Immunofluorescence also showed that fluoxetine upregulated p53 protein expression and promoted p53 trafficking from cytoplasm into nucleus (Fig. 6b). We next used p53 inhibitor PFT-α and found that PFT-α pretreatment could reverse the downregulation of p-mTOR and p62, and the upregulation of Atg7, beclin-1, and LC3II induced by fluoxetine (Fig. 6c). To confirm the critical role of p53 in regulating autophagy, we further cultured p53^−/−^ MEF cells. Interestingly, p53 knockout abolished fluoxetine-induced increase in LC3II/LC3I ratio and decrease in p62 expression (Fig. 6d). These results indicate that p53 presence is required for fluoxetine activated autophagy flux and fluoxetine promotes astrocytic autophagy in a p53-dependent mechanism.

### Fluoxetine produced cytoprotection via promoting autophagy in vitro

**Fig. 7:**
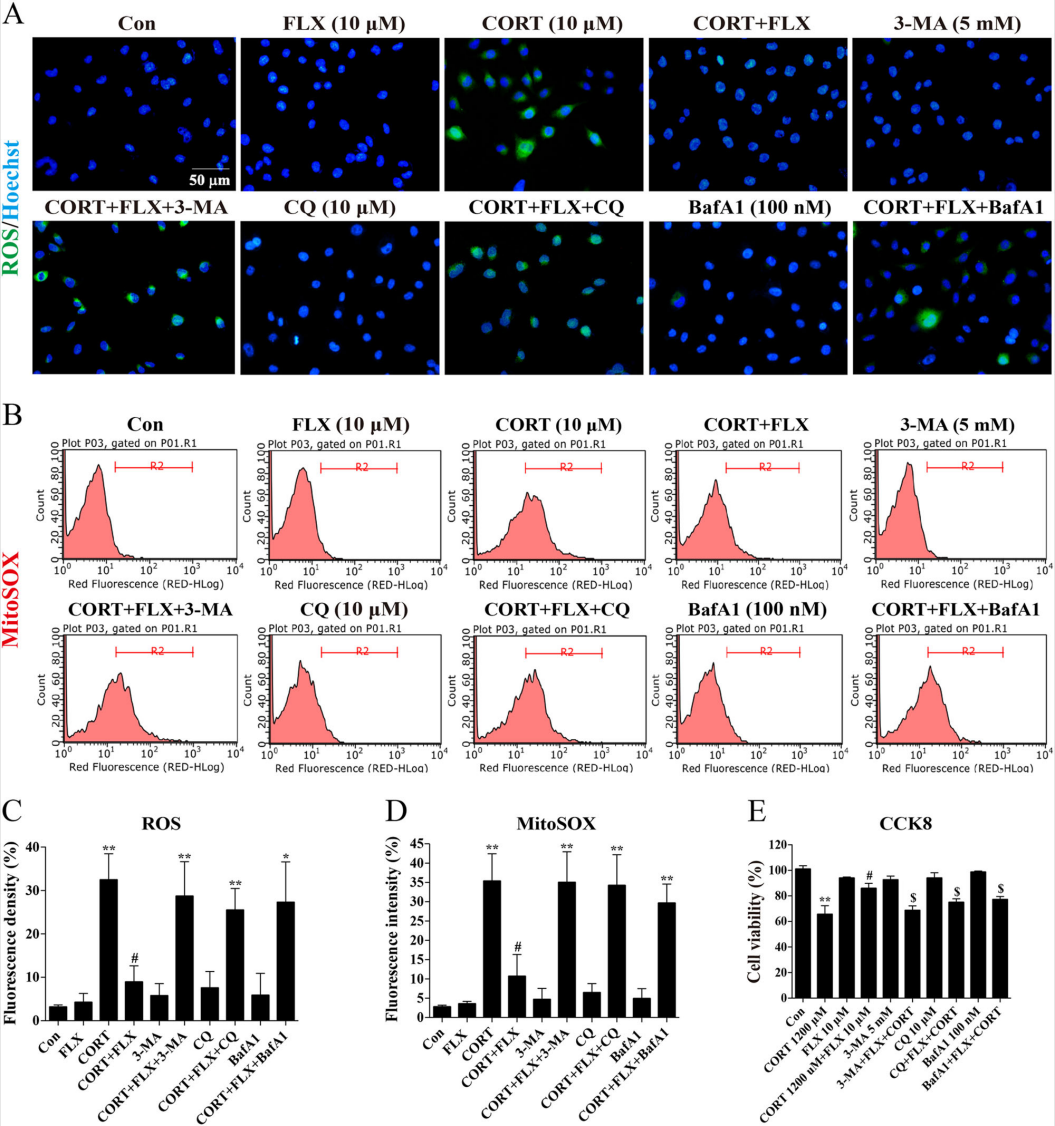
Fluoxetine produced cytoprotection on astrocytes via promoting autophagy. **a** Fluorescence images of intracellular ROS (green). Cells were stained with H_2_DCF-DA (20 μM) following fluoxetine treatment and captured under the fluorescence microscope. Scale bar: 50 μm. **b** Flow cytometry analysis of mitochondrial ROS stained by MitoSOX. **c**, **d** Quantitative analysis for fluorescence intensity of intracellular ROS and mitochondrial ROS, respectively. **e** CCK-8 assay for the effect of 3-MA, CQ, and BafA1 on fluoxetine-protected cell viability. Data are representative of three independent experiments and are expressed as mean ± SD. **p < 0.01 vs. Con group, ^#^*p* < 0.05, ^##^*p* < 0.01 vs. CORT group, ^$^*p* < 0.05 vs. CORT+FLX group

Impaired or nonfunctional mitochondria could release excessive ROS and lead to cellular damage. To evaluate the correlation between cytoprotection and proautophagic effect of fluoxetine, we applied different autophagy inhibitors to block mitophagy and used H_2_DCF-DA probes (20 μM) to label cytoplasmic ROS. As shown in Fig. 7a, c, ROS aggregated dramatically after 24 h stimulation of corticosterone and fluoxetine pretreatment decreased corticosterone evoked ROS accumulation. However, pretreatment of autophagy inhibitor 3-MA (5 mM), CQ (10 μM), or BafA1 (100 nM) could abolish the inhibitory effect of fluoxetine on corticosterone-induced ROS production. This result suggests that autophagy regulation contributes to the inhibitory effect of fluoxetine on ROS accumulation in astrocytes. Given that ROS derive predominantly from mitochondria, we further used MitoSOX to detect mitochondrial ROS. As shown in Fig. 7b, d, we analyzed fluorescence intensity by flow cytometry and found that fluoxetine reduced MitoSOX fluorescence intensity induced by corticosterone, while this effect was blocked by 3-MA, CQ, or BafA1. Subsequently, we employed EnoGeneCell^TM^ CCK-8 to assay cell viability. In agreement with above results, corticosterone (1.2 mM) inhibited cell viability and fluoxetine prevented astrocytes against cellular damage. When 3-MA, CQ, or BafA1 were used to block autophagy, cell viability was exacerbated significantly compared with that in corticosterone plus fluoxetine group (Fig. 7e). These findings indicate that fluoxetine eliminates excessive mitochondrial ROS and protects astrocytes via facilitating autophagy in vitro.

### Fluoxetine induced cytoprotection in vivo and ameliorated depressive-like behaviors via promoting autophagy

**Fig. 8:**
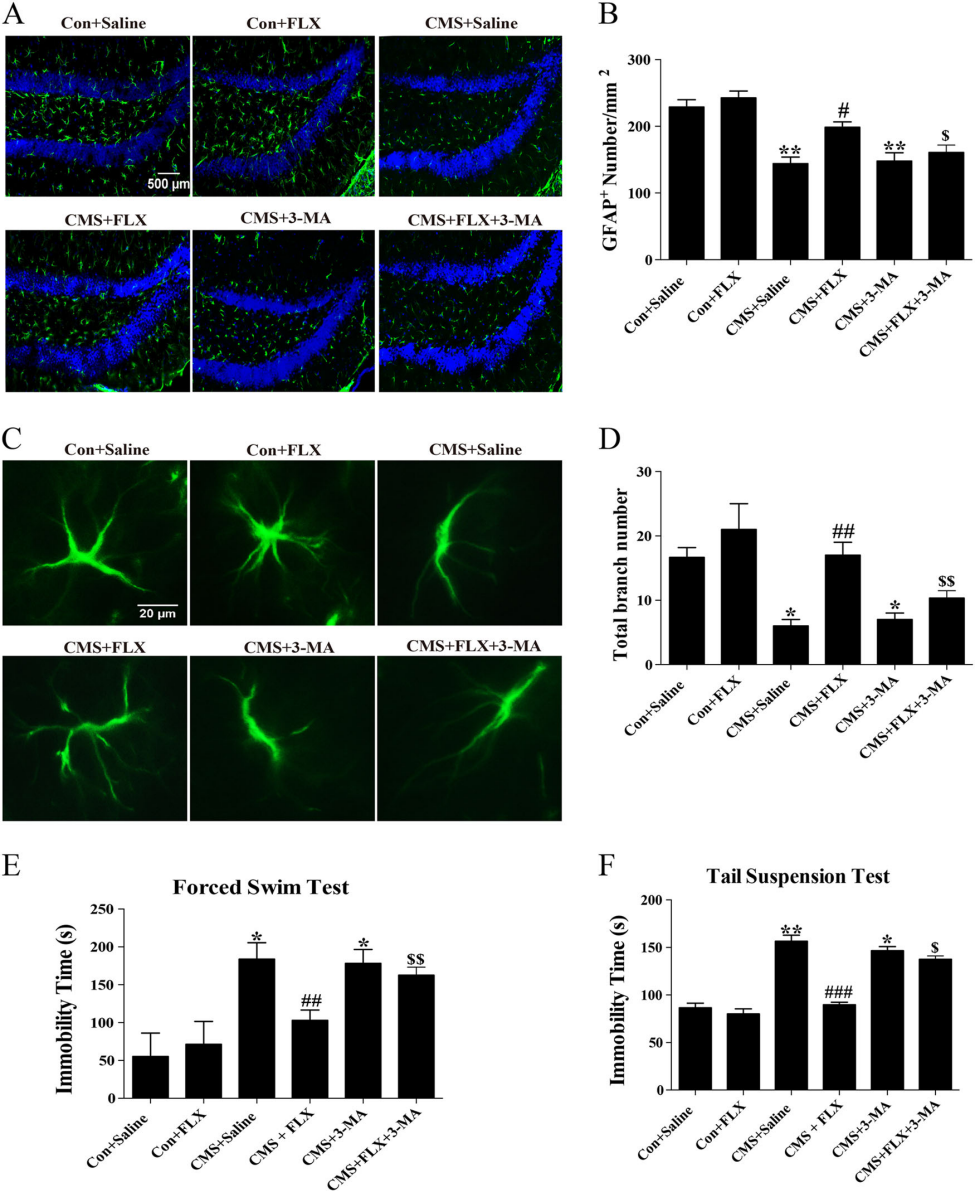
Fluoxetine protected astrocytes in vivo and ameliorated depressive-like behaviors via promoting autophagy. **a**, **b** Immunofluorescence images and cell counts of hippocampal GFAP^+^ astrocytes in fluoxetine and 3-MA treated CMS mice. Scale bar: 500 μm. **c**, **d** Immunofluorescence images of astrocytic morphology and quantitative analysis of total branch numbers in each group. Scale bar: 20 μm, *n* = 6. **e**, **f** Immobility time of fluoxetine and 3-MA treated CMS mice in FST and TST, respectively. *n* = 12, data are expressed as mean ± SD. *p < 0.05, **p < 0.01 vs. Con group, ^#^*p* < 0.05, ^##^*p* < 0.01, ^###^*p* < 0.001 vs. CMS group^, $^*p* < 0.05, ^$$^*p* < 0.01 vs. CMS+FLX group

To confirm the protective effect of fluoxetine on primary astrocytes in vitro, we also prepared CMS model and gave mice systemic administration of fluoxetine and 3-MA. We analyzed GFAP^+^ cell number and morphology by brain slice immunofluorescent staining and quantitative analysis of stereology. Notably, astrocytes in SGZ of control and fluoxetine alone treated mice displayed a vigorous shape, which have more and longer protuberances than those of CMS model mice. Fluoxetine treatment for 4 weeks alleviated the decrease in cell number (Fig. 8a, b) and astrocyte protuberance (Fig. 8c, d) of CMS mice, but this improvement could be abrogated by intracerebral stereotactic (30 μg, once) plus intraperitoneal (30 mg/kg, 4 weeks) injection of 3-MA (Fig. 8a–d). Therefore, we conclude that promoting autophagy is required for the protective effect of fluoxetine on astrocytes in CMS mouse model. Functionally, we further performed forced swim test and TST to assess the correlation between proautophagic and antidepressant effect of fluoxetine. We found that the immobility times were significantly prolonged in 3-MA treatment groups compared with those in fluoxetine-treated CMS groups in both tests (Fig. 8e, f). Consistently, fluoxetine extended CMS-reduced swimming time and climbing time in FST but 3-MA abolished this improvement of fluoxetine (Supplementary Fig. 3). The behavioral test results suggest that proautophagic effect and subsequent protection of astrocytes may contribute to the antidepressant role of fluoxetine in mouse model of depression.

## Discussion

Over the past decades, monoaminergic hypothesis had been regarded as the main pathogenesis of depression and acted as the biochemical basis for antidepressant drugs^31^. However, there are approximately one-third of depression patients who show poor reactivity to existing drugs clinically^8^. Fluoxetine, the first developed SSRI, is widely used in clinical treatment of major depression. In recent years, fluoxetine has been recognized to play multiple roles in antioxidation, anti-inflammation^19^, antitumor^20^, and neuroprotection beyond the serotonin hypothesis^18^. Emerging evidence shows that fluoxetine functions as a proautophagic agent in different cells. Although this has broken through our traditional cognition of fluoxetine, it remains unknown whether is fluoxetine-induced autophagy involved in the antidepressant effect in CMS model. In the present study, we found that fluoxetine treatment for 4 weeks alleviated depression-like symptoms while increasing LC3 expression and autophagosome formation in the hippocampus of CMS mice. It implies the possibility that regulating autophagy may be an alternative mode of action for fluoxetine besides its role in modulating serotonin. In short, we link autophagy with antidepressant effect of fluoxetine for the first time and further explore the significance of promoting autophagy in CMS model.

Of all the neural cells in the brains, astrocytes rank the first place in the number and distribution, which exist between neurons and capillary. They contribute to brain homeostasis in a couple of ways^32^. Notably, postmortem analysis of the prefrontal cortex and hippocampus demonstrated a decreased number of astrocytes in patients suffering from major depression^33^ and selectively acute injury to astrocytes is adequate to induce depressive-like behaviors^34^. Thus, protecting astrocytes to maintain neuronal survival and function may be an effective approach in the action of antidepressants and potential treatment strategy for depression. Whether proautophagic effect of fluoxetine could be beneficial to the protection of astrocytes in depression is not understood totally. We cultured primary astrocytes in vitro to clarify the impact of fluoxetine-induced autophagy on astrocytic survival. In line with what we observed in CMS mice in vivo, fluoxetine prevented astrocytes against corticosterone-induced cell death and this effect was blocked by autophagy inhibitor, 3-MA. In addition to increasing synaptic cleft 5-HT levels, the antidepressant effect of fluoxetine is presumably derived from relieving astrocyte pathological damage, which is mediated by promoting autophagy.

Mitochondria are essential organelles that regulate cellular homeostasis and cell survival^29^ and it is proposed that impaired mitochondrial function contributes to the pathophysiology of MDD^35,36^. Mitophagy is a process that selectively removes disrupted mitochondria through autophagy^37^. Autophagy and mitophagy are considered as emerging mechanisms in the action of some antidepressants^38–40^. In the present study, we found fluoxetine enhanced Parkin translocating from cytoplasm to mitochondria to degrade TOMM20 and induced a decrease in mitochondrial TOMM20 protein level. It means that fluoxetine promotes astrocytic mitophagy flux and increases the clearance of damaged mitochondria in corticosterone-treated cell model. As a result, fluoxetine reduced mitochondria-derived ROS accumulation, which consequently attenuated cell death caused by mitochondrial damage. Therefore, we propose a new hypothesis in astrocytes by which FLX promotes autophagy to eliminate damaged mitochondria and to reduce cell death, and finally ameliorates pathological changes in hippocampal astrocytes in CMS model of depression. Blockage of autophagy could partially abolish the antidepressant effect of FLX, suggesting that regulation of 5-HT transmission in neurons and promotion of autophagy in astrocytes may exist simultaneously in antidepressant role of FLX.

The mammalian cell “gatekeeper” p53 belongs to one of the tumor suppressor families that are most common regulators of apoptosis and autophagy^41^. Experimental evidence convincingly indicates that p53 can act as either an activator or an inhibitor of autophagy depending on its subcellular localization and its mode of action^42^. Stress-induced activation of p53 protein nuclear translocation is able to stimulate proautophagic action by transcriptional upregulation of autophagy related gene. In contrast, cytoplasm-localized physiological p53 protein has an inhibitory effect on autophagy via inhibiting the AMP-dependent kinase, a positive regulator of autophagy, and activating mTOR^43^. Interestingly, we found fluoxetine promoted autophagy flux, accompanied by the nuclear translocation of p53 in astrocytes. Either p53 inhibitor PFT-α or p53 knockout could abolish the inhibition of mTOR pathway and subsequent autophagy activation induced by fluoxetine. Our previous study has also revealed that p53 is a critical target protein for fluoxetine in protecting neurons against IL-1β-induced apoptosis^30^. As expected, p53 is required for the proautophagic effect of fluoxetine in mouse astrocytes. The exact mechanism for nuclear translocation of p53 regulated by fluoxetine is in the need of further exploration.

To finally confirm the impact of autophagy on pathology and depression-like behavior, we treated mice with intracerebroventricular injection of autophagy inhibitor 3-MA in vivo. The protection of hippocampal astrocytes by fluoxetine covers the restoration in both of cell number and morphology in CMS model. Once using 3-MA, a marked reduction in number, volume, and protuberance of astrocytes in the presence of fluoxetine treatment was found. Most importantly, inhibiting autophagy by 3-MA could abrogate the improvement of fluoxetine on CMS-induced depressive behaviors, evidenced by the prolonged immobility time in FST and TST. These results further support that autophagy activation is an indispensable mechanism for the roles of fluoxetine in protecting astrocytes and relieving depressive symptoms.

In conclusion, our present study unveils a novel protective mechanism in astrocytes by which fluoxetine promotes autophagy to eliminate damaged mitochondria and to reduce cell death, and finally ameliorates pathological changes in hippocampal astrocytes in CMS model of depression (Supplementary Fig. 4). Hopefully, this work gives us insights into a novel approach to treat depression depending on astrocytes, and provides a promising molecular target for the development of antidepressant drugs besides regulating neurotransmitters.

## Supplementary information

Supplementary figure legends

Supplementary Figure 1

Supplementary Figure 2

Supplementary Figure 3

Supplementary Figure 4

Supplementary Figure 5
