# Supplementary material for: The effect of fluoxetine on astrocyte autophagy flux and injured mitochondria clearance in a mouse model of depression
Source: Cell Death Dis. 2019 Aug 2;10(8):577. doi: 10.1038/s41419-019-1813-9 (PMC6675792; doi:10.1038/s41419-019-1813-9)
Supplement: Supplementary file 1 — Supplementary figure legends [file 41419_2019_1813_MOESM1_ESM.doc]

**Supplementary figure legends:**

**Supplementary Fig. 1**

(A) The swimming time in forced swimming test and (B) the climbing time in forced swim test. n=6, data are expressed as mean±SD. ^**^ p<0.01 vs. Con group, ^#^ p<0.05 vs. CMS group.

**Supplementary Fig 2**

(A) Concentration of CORT in both hippocampus and (B) plasma of CMS model mice by ELISA assay. n=6, data are expressed as mean±SD. ^**^ p<0.01 vs. Con group, ^##^ p<0.01 vs. CMS group.

**Supplementary Fig 3**

(A) The swimming time in forced swimming test and (B) the climbing time in forced swim test. n=6, data are expressed as mean±SD. ^**^ p<0.01 vs. Con group, ^#^ p<0.05 vs. CMS group, ^$^ p<0.05 vs. CMS+FLX group.

**Supplementary Fig 4**

**Schematic illustration for the pro-autophagic and cytoprotective effects of fluoxetine on hippocampal astrocytes in a mouse model of depression** Solid arrow represents promotion, crosshair represents inhibition, dotted arrow represents local enlargement.

**Supplementary Fig 5**

Uncropped Western blotting images for review only.
