# Supplementary figures and images for: The effect of fluoxetine on astrocyte autophagy flux and injured mitochondria clearance in a mouse model of depression
Source: Cell Death Dis. 2019 Aug 2;10(8):577. doi: 10.1038/s41419-019-1813-9 (PMC6675792; doi:10.1038/s41419-019-1813-9)

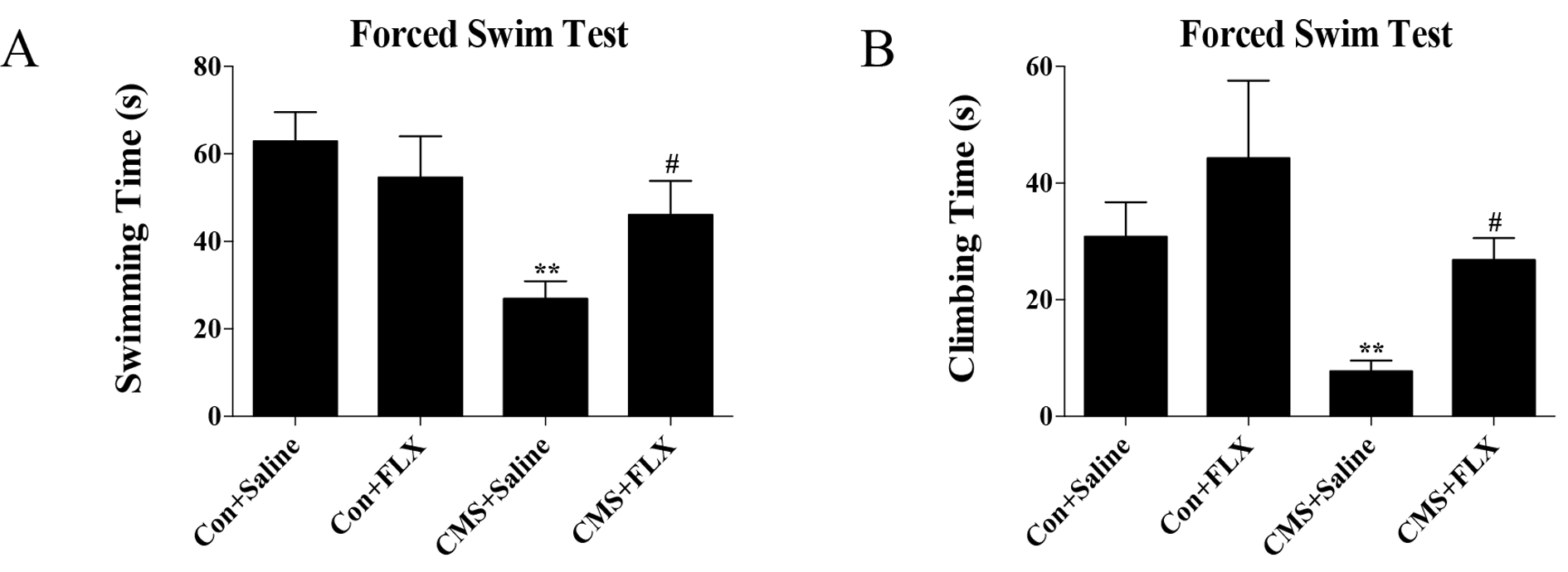

Supplement: Supplementary file 2 — Supplementary Figure 1 [file 41419_2019_1813_MOESM2_ESM.tif]

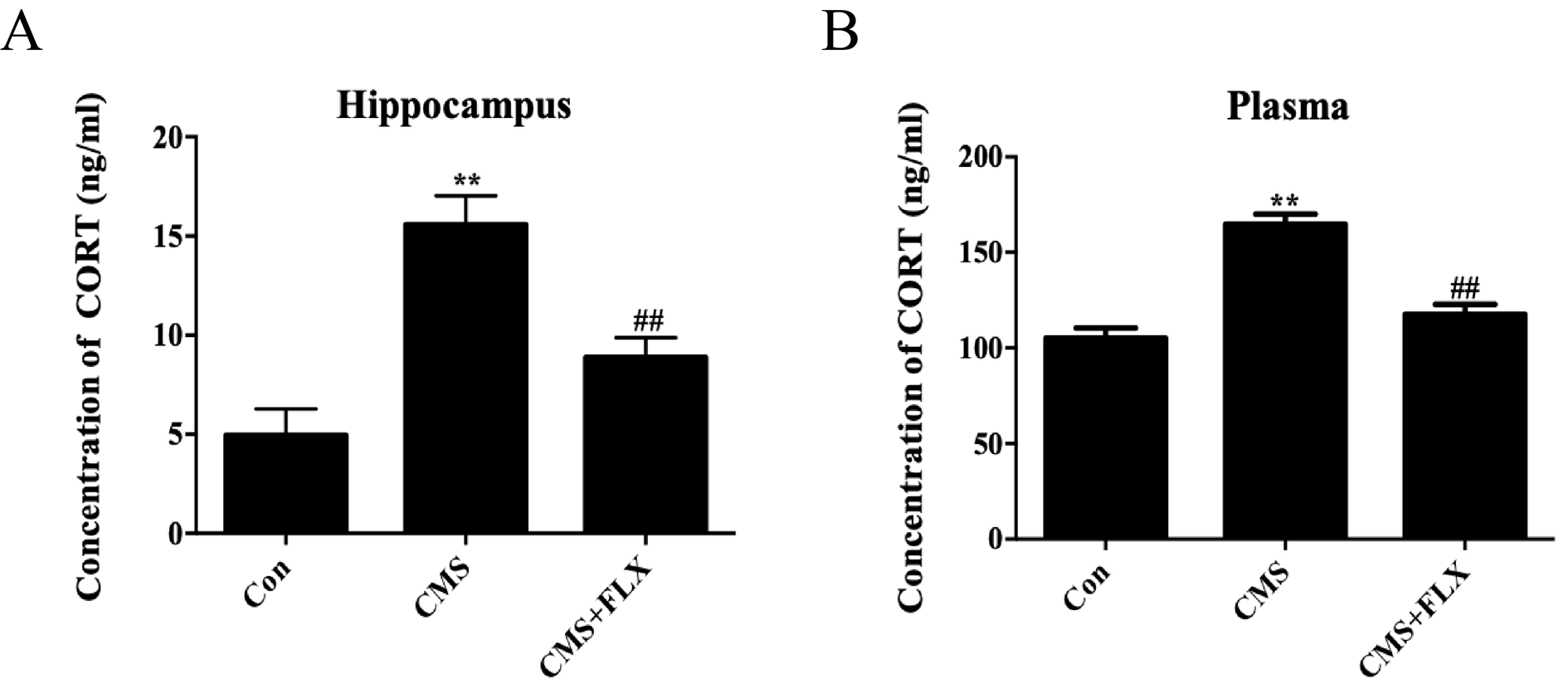

Supplement: Supplementary file 3 — Supplementary Figure 2 [file 41419_2019_1813_MOESM3_ESM.tif]

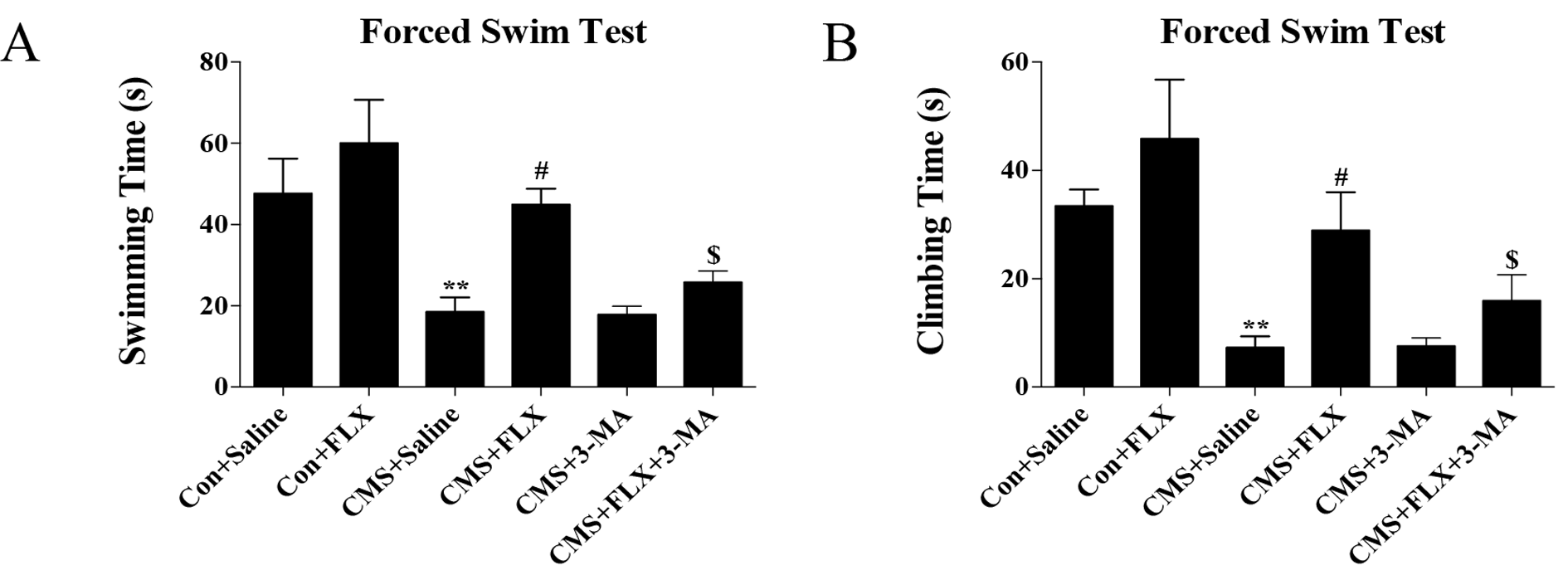

Supplement: Supplementary file 4 — Supplementary Figure 3 [file 41419_2019_1813_MOESM4_ESM.tif]

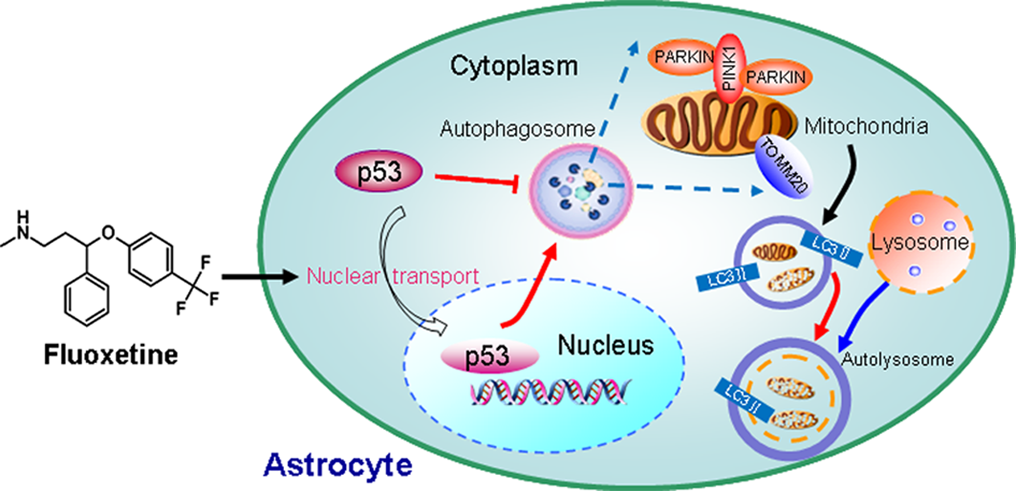

Supplement: Supplementary file 5 — Supplementary Figure 4 [file 41419_2019_1813_MOESM5_ESM.tif]

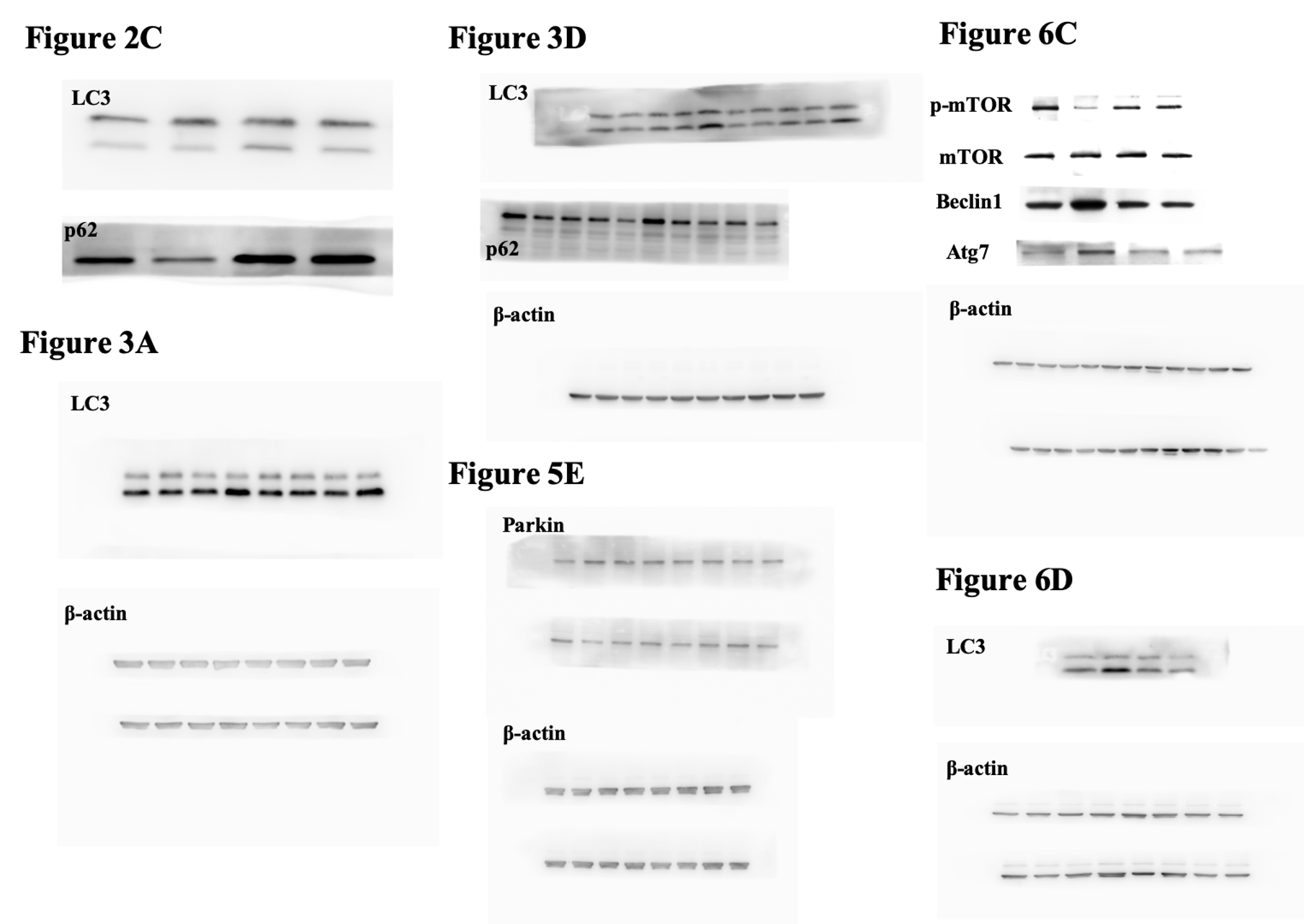

Supplement: Supplementary file 6 — Supplementary Figure 5 [file 41419_2019_1813_MOESM6_ESM.tif]
